# An enzyme in disguise

**DOI:** 10.1107/S2052252520002481

**Published:** 2020-02-29

**Authors:** Anders Liljas

**Affiliations:** a Biochemistry and Structural Biology, Lund University, Lund, Sweden

**Keywords:** catalytic metal ions, copper–carbonic anhydrase II, carbonic anhydrases, nitrite reductases

## Abstract

The enzyme carbonic anhydrase binds its zinc ion by three histidine residues in a similar manner to the way copper is bound to nitrite reductase. This remote similarity has now been shown to be real [Andring *et al.* (2020). *IUCrJ*, **7**, 287–293]. A carbonic anhydrase with two bound copper ions is also a nitrite reductase.

Carbonic anhydrase (CA) is a very well known enzyme that was discovered more than 80 years ago (Meldrum & Roughton, 1933 ▸). It is responsible for catalyzing the conversion of carbon dioxide to bicarbonate in all cells and the opposite conversion in the lungs. Several forms of the enzyme have been identified as well as numerous isoenzymes of the human α form (Lomelino *et al.*, 2018 ▸). The different forms are found in different species and isoenzymes are located in various human cell types. An exciting discovery is that isoenzymes IX and XII are associated with cancer cells and are probably important for cancer growth (Pastorekova *et al.*, 2007 ▸, 2008 ▸).

All carbonic anhydrases are metal enzymes, with zinc as the prime metal. In the first structure of carbonic anhydrase (αCAII), a zinc ion was seen bound to three histidine residues at the core of the active site (Liljas *et al.*, 1972 ▸). In studies of nitride reductase it was observed that the metal, in this case copper, was bound at two sites, type 1 and type 2. The type 2 copper is bound with the same configuration as in CA, by three histidines (Strange *et al.*, 1995 ▸). Based on the similarity in substrate structure, Aamand *et al.* (2009 ▸) investigated whether CA could reduce nitrite. They found this to be the case but an explanation could not be envisioned. Andring *et al.* (2018 ▸) also made a thorough study but could not repeat the results of Aamand *et al.* (2009 ▸).

Among the extensive crystallographic investigations of CA some have involved changing the metal. Håkansson *et al.* (1994 ▸) observed that when the zinc ion was exchanged for copper, one additional copper site could be observed. This was in the floppy N-terminal region containing two His residues. In addition, they observed a presumed diatomic molecule bound to the copper at the classical active site. Similarly, Ferraroni *et al.* (2018 ▸) observed two copper atoms bound as well as the diatomic molecule at the active site. Neither of these studies made any connection to nitrite reductase.

In the current issue of **IUCrJ**, Andring *et al.* (2020 ▸) have been able to unravel the mystery. Carbonic anhydrase (αCAII) binds zinc as well as copper physiologically. The zinc-bound enzyme catalyzes the reversible conversion between carbon dioxide and bicarbonate. The same enzyme with two bound copper ions functions as a nitrite reductase. The classical active site for copper is site 2 where the reduction takes place. The diatomic ‘molecule’ seen in several studies is here interpreted as NO_2_^−^ that has somehow been generated. At the other site (1) at the N-terminus copper binds in a manner that imitates binding to a porphyrin ring. The role of this site is to reduce the copper in the active site. The flexible His64 between the two sites, as well as a number of bound water molecules, are probably engaged in electron transport between the two sites (Fig. 1) ▸.

This discovery, which has eluded the field for so long, is both remarkable and unusual. The long-standing assumption of a relatively simple role for the enzyme carbonic anhydrase has missed its second role as a more complex nitrite reductase. This discovery should lead to further experiments and research to better elucidate the role of this enzyme and decipher how these new insights could be applied in medical treatments.

## Figures and Tables

**Figure 1:**
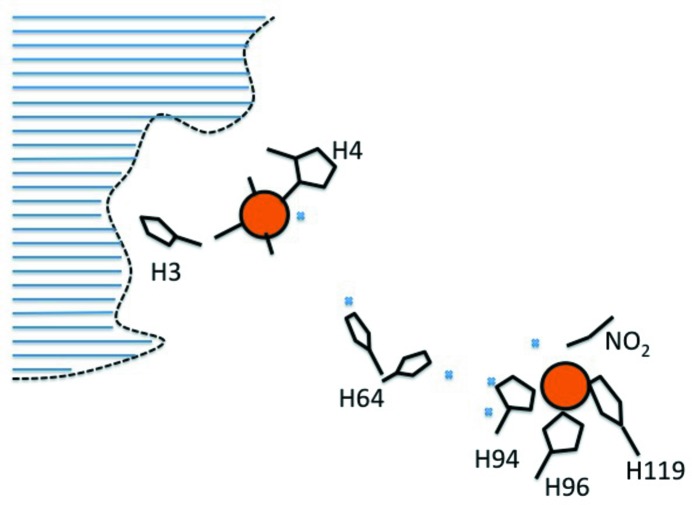
The two copper ions (red–brown) bound to the surface of human α-carbonic anhydrase II. The copper to the right is situated at the classical active site where the zinc ion is normally bound by three histidine ligands (His94, His96 and His119). An NO_2_^−^ ion is bound at this copper. The left copper is the second metal site where copper can bind in a square-planar nitro­genous setting like in a porphyrin. His64, which is found in two conformations, and a number of observed water molecules (blue dots), can facilitate the transport of electrons between the two metals.

## References

[bb1] Aamand, R., Dalsgaard, T., Jensen, F. B., Simonsen, U., Roepstorff, A. & Fago, A. (2009). *Am. J. Physiol. Heart Circ. Physiol.* **297**, H2068–H2074. 10.1152/ajpheart.00525.2009 19820197

[bb2] Andring, J. T., Kim, C. U. & McKenna, R. (2020). *IUCrJ*, **7**, 287–293. 10.1107/S2052252520000986 PMC7055381 32148856

[bb3] Andring, J. T., Lomelino, C. L., Tu, C., Silverman, D. N., McKenna, R. & Swenson, E. R. (2018). *Free Radic. Biol. Med.* **117**, 1–5. 10.1016/j.freeradbiomed.2018.01.015 29355738

[bb4] Ferraroni, M., Gaspari, R., Scozzafava, A., Cavalli, A. & Supuran, C. T. (2018). *J. Enz. Inhib. Med. Chem.* **33**, 999–1005. 10.1080/14756366.2018.1475371 PMC6010096 29806484

[bb5] Håkansson, K., Wehnert, A. & Liljas, A. (1994). *Acta Cryst.* D**50**, 93–100. 10.1107/S0907444993008790 15299481

[bb6] Liljas, A., Kannan, K. K., Bergstén, P. C., Waara, I., Fridborg, K., Strandberg, B., Carlbom, U., Järup, L., Lövgren, S. & Petef, M. (1972). *Nature New Biol.* **235**, 131–137. 10.1038/newbio235131a0 4621826

[bb7] Lomelino, C. L., Andring, J. T. & McKenna, R. (2018). *Int. J. Med. Chem.* **2018**, 1–21. 10.1155/2018/9419521 PMC6158936 30302289

[bb8] Meldrum, N. U. & Roughton, F. J. W. (1933). *J. Physiol.* **80**, 113–142. 10.1113/jphysiol.1933.sp003077 PMC1394121 16994489

[bb9] Pastorekova, S., Kopacek, J. & Pastorek, J. (2007). *Curr. Top. Med. Chem.* **7**, 865–878. 10.2174/156802607780636708 17504131

[bb10] Pastorekova, S., Zatovicova, M. & Pastorek, J. (2008). *Curr. Pharm. Des.* **14**, 685–698. 10.2174/138161208783877893 18336315

[bb11] Strange, R. W., Dodd, F. E., Abraham, Z. H., Grossmann, J. G., Brüser, T., Eady, R. R., Smith, B. E. & Hasnain, S. S. (1995). *Nat. Struct. Mol. Biol.* **2**, 287–292. 10.1038/nsb0495-287 7796265

